# Iodide of Strontium in Cardiac and Other Affections

**Published:** 1893-08

**Authors:** 


### IODIDE OF STRONTIUM IN CARDIAC AND OTHER AFFECTIONS.

BY DRS. LABORDE & MALBEC.

(Translated from the Tribune Medicale, 15th December, 1892.)

The clinical application of Iodide of Strontium has been retarded on account ot the extreme difficulty which was first experienced in procuring a chemically pure and at the same time a stable salt.

The facility with which commercial Iodides give up their Iodine, and the frequent presence of the toxic Iodate, has on more than one occasion been commented upon at the French Academy of Medicine, and it has been found necessary to issue a caution against the Strontium Iodide in any other than the crystalized form.

The most practical mode of manufacture, is to decompose a solution of Iodide of Iron by Sulphide of Strontium. The resulting solution is rapidly filtered in an atmosphere deprived of oxygen, and the Sulphide of Iron separated ; the solution of Iodide of Strontium is then evaporated, crystallized and recrystallized several times, in order to obtain a perfectly pure salt, unchanged by the action of the air and light.

Iodide of Strontium crystalizes in hexagonal tables containing six molecules of water.

The question of absolute purity is very important, otherwise it rapidly decomposes, when exposed to the air.

The problem of producing a stable and crystallized salt, without which exact physiological observation was hitherto impossible, has been solved by Paraf-Javal. From the investigations reported to the Biological Society, May 28th, 1892, it was shown that the action of Iodide of Strontium on the circulation of animals is similar to that of Iodide of Potassium.

Practical clinical experience with Strontium Iodide (Paraf-Javal), has fully confirmed the supposition that the Iodide of Strontium would present the same advantage over the Potassium salt as its Bromide analogue, and a series of observations was undertaken by one of us, on account of the non-poisonous character of the salt as compared with Potassium Iodide.

Observation 1. A lady *æt.*50, with chronic endocarditis, and an enfeebled action of the heart, suffered from considerable dyspnœa together with symptoms of angina pectoris. The patient was treated for some time with Iodide of Potassium which gave considerable relief, but she was unable to continue its use any longer on account of the gastric-irritation to which it gave rise. The *standatd solution of Strontium Iodide*, (30 grains to the ounce) was therefore substituted, beginning with one tablespoonful, subsequently increased to two tablespoonfuls daily. The change proved eminently satisfactory, and within twenty-four hours a marked improvement in the symptoms was observed and the amelioration was maintained by its continued use.

Observation 2. A young professor, affected with a cardio-pulmonary affection, characterized symptomatically by attacks of depression at times, taking the form of angina pectoris with a state of incomplete syncope. The heart presented signs of chronic endocarditis with a predominant determination on the left side of the auriculo ventricular orifices, which is the seat of an *insufficiency* shown by a rude *souffle* snbsisting at the left and at the point of normal *btuit*, as well as by a marked systolic tendency of the radial pulsations.

The patient had been benefited by Iodide of Potassium but was unable to continue its use, even in small doses of from 15 to 20 grains daily, without gastric derangements, which prevented the proper functions of digestion and alimentation. He was very weak, discouraged, and a prey to attacks of præcordial agony. A tablespoonful of the *standard solution of Iodide of Strontium* was administered, and increased later to a tablespoonful and a half daily. The effect was most remarkable, and in a few days he was able to resume his occupation and gradually the functional phenomena to which he was subject disappeared, the nutritive functions improved, and a fair condition of health is now established.

Observation 3. Our patient is a talented artist aged 50 years, who has suffered for a long time with an endocarditis, localized on the left side of the heart, and of the auriculo ventricular orifice with arterio-sclerosis and a complication with considerable gastric ectasis, and rebellious dyspeptic conditions. He is subject to attacks of depression which recently have become of extreme gravity, and which affect his respiration, causing a loss of consciousness and a fear of death. Small doses of Iodide of Potassium had previously helped him but had to be abandoned. A tablespoonful of Iodide of Strontium solution was prescribed, and after the third day there was considerable improvement of the cardiac functions and the dose was increased to two tablespoonfuls daily, this was continued for a month; the attacks of oppression and præcordial agony had disappeared, and there was considerable improvement in nutrition.

Observation 4. The patient is a powerful man of 40 years old, who is subject to frequent attacks of neuro-muscular rheumatism, which manifested themselves on the heart by phenomena of pain, dyspnœa and præcordial oppression. Auscultation demonstrated a commencement of endocarditis and particularly of the aortic ventricular orifice. The patient had never been submitted to any special treatment for this cardiac complication. Two tablespoonfuls of *solution Iodide of Strontium* (Paraf-Javal) were administered, which was perfectly tolerated. Under its influence the dyspnœa and oppression disappeared entirely in the course of two months.

Our conclusions upon these and other observations show that Iodide of Strontium by its favorable and rapid action in morbid cardiac and cardio-pulmonary troubles, are that this salt is superior and preferable to Iodide of Potassium, for in no case has any symptom of intolerance, such as cephalalgia, coryza, with nasal and salivary hypersecretion, cutaneous eruptions, etc., been noticed.

It is therefore permissible, on the physiological and clinical evidence, to consider Iodide of Strontium as an excellent substitute for the Iodide of Potassium, with this considerable advantage, that, although the dose is the same as that of the potassium salt, it may be increased when necessary without having to fear the intolerance, which would almost certainly follow larger doses of the latter.

The indications for the use of Iodide of Strontium are of course those of Iodide of Potassium, in which are included cardiac and cardio-vascular affections due to arterio-sclerosis; lesions of the myocardium and intra-cardiac orifices, asthma, angina pectoris, and in chronic and muscular rheumatism and gout, it is also indicated in the treatment of that large class of skin affections recognized to be amenable to Iodine, also in inflammations of a strumous type.

It may be advantageously employed in inflammatory conditions, such as pleurisy, peritonitis either simple or tuberculous, pericarditis, certain forms of pneumonia and in pulmonary emphysema.

This medicament is clearly indicated moreover in the treatment of enlarged glands such as the amygdalæ, in mammary hypertrophy and in enlargement of the uterus or prostate.

Iodide of Strontium (Paraf-Javal) does not set up gastric irritation or cause palpitation of the heart. It is speedily eliminated by the kidneys, while in its various applications the special action of the Iodide is supplemented by the beneficial effects which Strontium has been proved to exercise on the functions of nutrition.

